# Partial Situs Inversus Complicating the Surgical and Diagnostic Course of a Duodenal Switch Revision: A Case Report

**DOI:** 10.7759/cureus.117314

**Published:** 2026-10-01

**Authors:** Adelina Balidemaj, Naazpreet Harika, Mohammed Arain

**Affiliations:** 1 Clinical Sciences, Saint James School of Medicine, Chicago, USA; 2 General Surgery, UChicago Medicine AdventHealth, La Grange, USA; 3 Clinical Sciences, St. George's University School of Medicine, True Blue, GRD

**Keywords:** bariatric surgery, duodenal switch, enterocutaneous fistula, intra-abdominal abscess, partial situs inversus, revisional surgery, situs inversus

## Abstract

Situs inversus is a rare congenital disorder of left-right organ orientation characterized by mirror-image transposition of some or all visceral structures. While situs inversus totalis involves the transposition of both thoracic and abdominal organs, partial situs inversus involves only a subset of organs. The condition is frequently asymptomatic but can complicate diagnostic evaluation and surgical management when anatomic relationships differ from expected landmarks. We present a 44-year-old woman with known partial situs inversus involving the intestinal tract, without the transposition of the major thoracic or abdominal organs, and a history of duodenal switch bariatric surgery. Her postoperative course was complicated by small bowel obstruction, intra-abdominal abscesses, bowel perforation, enterocutaneous fistula formation, and polymicrobial infection requiring multiple operative and percutaneous interventions. She underwent two exploratory laparotomies and abdominal washout procedures, including the revision of her duodenal ileostomy, the extensive lysis of adhesions, and appendectomy. Her subsequent management involved multiple drain placements, prolonged antimicrobial therapy, nutritional support, and the management of a persistent right-sided abdominal drain with biliary output concerning for a duodenocutaneous fistula. During her prolonged course, the recognition of her altered intestinal anatomy remained an important consideration in her complex postoperative management. Although the severity of her postoperative complications cannot be attributed directly to situs inversus, the condition added complexity to an already challenging revisional surgical course. This case highlights the importance of recognizing and clearly documenting altered intestinal anatomy throughout prolonged, multi-stage abdominal surgical care.

## Introduction

Situs inversus is a rare congenital disorder resulting from abnormal left-right orientation during embryologic development. In situs inversus totalis, the thoracic and abdominal organs demonstrate a mirror-image arrangement relative to normal anatomy, whereas partial forms involve only a subset of organs. The reported prevalence of situs inversus is approximately one in 10,000 individuals, although the frequency varies depending on the specific anatomic phenotype [1]. Most individuals with situs inversus are asymptomatic, and the condition may remain undiagnosed until incidental imaging, surgery, or evaluation for an unrelated condition [1,2].

Although situs inversus itself generally does not require treatment, abnormal organ orientation can create important challenges during diagnostic and therapeutic procedures. Clinicians may encounter unexpected organ positions, altered vascular and gastrointestinal anatomy, and unfamiliar relationships between structures during surgery or interventional procedures [1,2]. These differences can complicate the interpretation of imaging studies and require the modification of standard operative approaches. In acute surgical conditions, altered anatomy may also produce atypical symptom localization, potentially contributing to delayed or incorrect diagnosis [3,4].

The surgical literature concerning situs inversus has largely focused on isolated procedures, particularly appendectomy and cholecystectomy. In patients with situs inversus totalis, appendicitis may present with left-sided rather than right-sided abdominal pain, while cholecystitis may present with left upper quadrant symptoms. Preoperative imaging and the careful recognition of abnormal anatomy are therefore emphasized to facilitate appropriate diagnosis and operative planning [3,4]. Surgical procedures can generally be performed safely when the altered anatomy is recognized, although modifications to operative positioning, port placement, and surgical technique may be required [1,5].

Bariatric surgery presents additional considerations because of the extensive manipulation of the gastrointestinal tract and the need to account for altered anatomy during both primary and revisional procedures. A systematic review of bariatric and metabolic surgery in patients with situs inversus identified a limited number of reported cases and emphasized the technical challenges created by mirror-image anatomy, particularly during laparoscopic procedures [6]. However, there is limited literature describing situs inversus in the setting of a prolonged, multi-stage revisional bariatric surgical course complicated by intra-abdominal infection, fistula formation, repeated drain procedures, and nutritional compromise.

We present a case of partial situs inversus involving the intestinal tract, without the transposition of the major thoracic or abdominal organs, complicating the diagnostic and surgical management of a patient undergoing a complex duodenal switch revision. The case highlights the importance of recognizing altered intestinal anatomy during subsequent imaging, reoperations, drain management, and other stages of a prolonged surgical course.

## Case presentation

A 44-year-old woman with known partial situs inversus involving the intestinal tract and a history of duodenal switch bariatric surgery for obesity subsequently developed a complicated postoperative course requiring multiple surgical and interventional procedures. She presented to an outside hospital with persistent nausea and vomiting and no bowel movement for approximately two weeks. She denied significant abdominal pain or fever but reported substantial abdominal bloating. Initial evaluation demonstrated a partial small bowel obstruction, during which a bowel perforation related to her recent bariatric procedure was identified. Given her complex surgical history and need for higher-level surgical management, she was transferred to a tertiary care center. At the receiving institution, she underwent exploratory laparotomy with revision of the duodenal ileostomy, extensive lysis of adhesions, and appendectomy. Although her partial situs inversus did not require a fundamentally different operative technique, the recognition of the altered intestinal orientation remained an important anatomic consideration during the procedure. Her postoperative course was complicated by intra-abdominal abscess formation requiring two washout surgeries and frank biliary output from a right-sided abdominal drain, raising concern for a leak involving the duodenal stump. Repeat computed tomography (CT) imaging demonstrated increasing loculated intra-abdominal fluid collections, prompting the placement of multiple percutaneous drains for source control (Figure 1).

**Figure 1:**
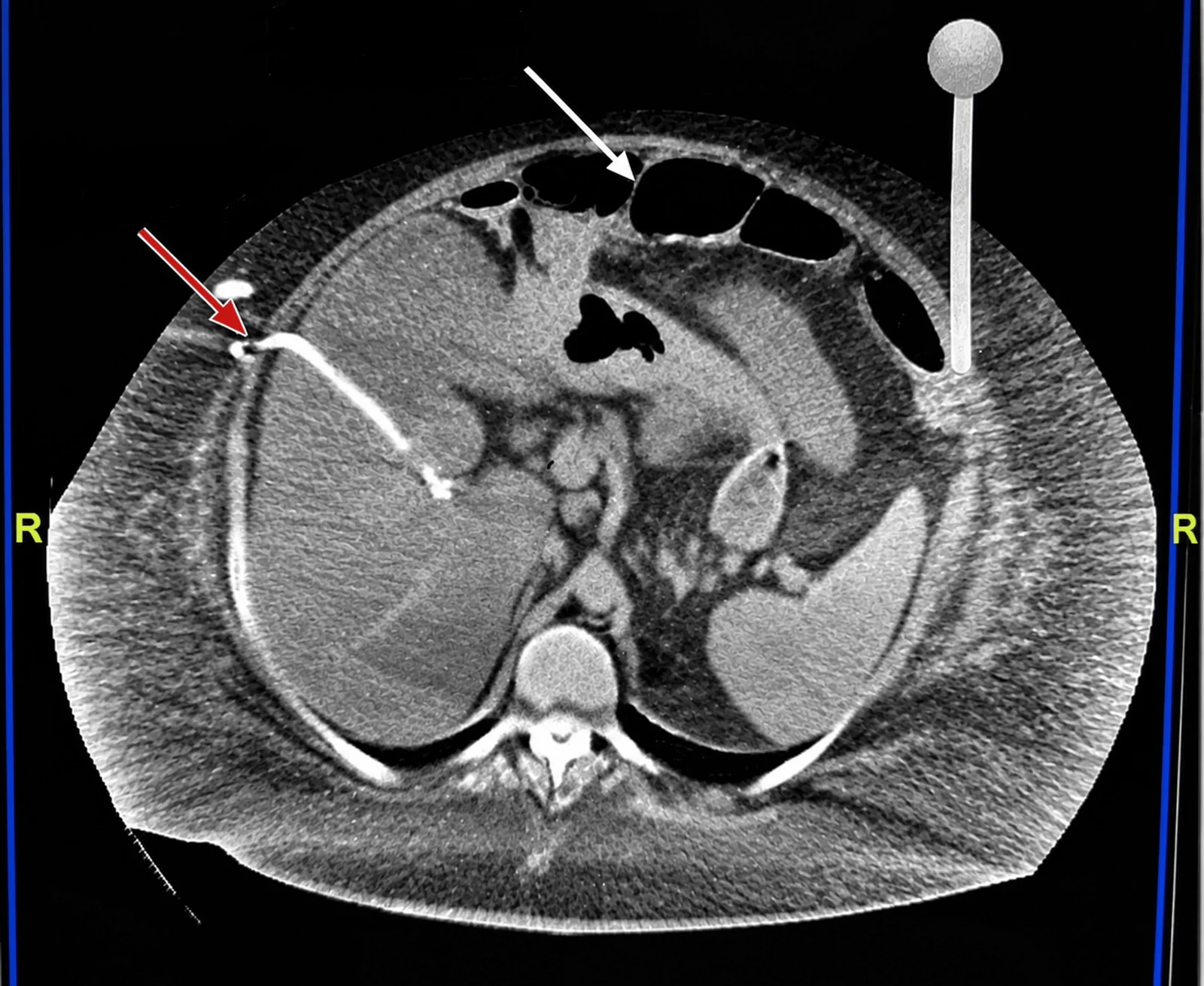
Postoperative abdominal computed tomography demonstrating intra-abdominal changes and percutaneous drain placement. Axial contrast-enhanced computed tomography (CT) image of the abdomen demonstrating prominent dilated, gas-filled bowel loops (white arrow) and an indwelling percutaneous drainage catheter (red arrow).

She subsequently developed an enterocutaneous fistula with persistent biliary output from the right-sided abdominal drain, raising concern for a possible duodenocutaneous fistula related to a duodenal blowout. Her postoperative course was further complicated by recurrent polymicrobial intra-abdominal infections requiring treatment. A large abdominal surgical wound also developed, requiring ongoing wound care. She required nutritional support, including bowel rest and parenteral nutrition, followed by the gradual reintroduction of enteral nutrition through a gastrostomy tube. She also developed *Clostridioides difficile* infection, for which she received treatment. As her clinical condition improved, enteral nutrition was gradually advanced as tolerated, while oral intake was initially limited to full liquids.

On reassessment, her abdominal wound was approximately 95%-99% healed, with only a small area of persistent skin separation and no evidence of active wound infection. The right-sided abdominal drain continued to produce considerable amounts of clear bile, while the left-sided drain had minimal output. The left-sided abdominal drain was removed at bedside because of its minimal output and lack of a significant residual fluid collection on imaging. The pigtail catheter and retaining suture were removed without complication, bleeding, or subsequent drainage from the site. The right-sided abdominal drain was retained because of continued biliary output and concern for a persistent duodenocutaneous or enterocutaneous fistulous communication. The right-sided drain had been temporarily clamped and was tolerated without significant discomfort or recurrence of symptoms.

Enteral feeding was gradually liberalized, and she tolerated advancement without recurrent nausea or vomiting. At most recent follow-up, the patient remained hemodynamically stable and afebrile. Intermittent abdominal discomfort remained her primary ongoing complaint. Her overall course was also complicated by morbid obesity, asthma with a history of obstructive sleep apnea, depression and anxiety symptoms, a sacral wound, and protein-calorie malnutrition, which were managed as part of her multidisciplinary care.

## Discussion

This case illustrates the diagnostic and surgical considerations associated with partial situs inversus during an already complex revisional abdominal surgical course. Partial situs inversus is generally asymptomatic in isolation, but altered visceral orientation can complicate diagnostic reasoning, imaging interpretation, and surgical procedures when clinicians encounter anatomy that differs from expected relationships [1,2].

Most published literature regarding situs inversus and surgery describes isolated procedures, particularly appendectomy and cholecystectomy. Appendicitis in patients with situs inversus totalis may present with left-sided abdominal pain rather than the classic right lower quadrant presentation. A systematic review of published cases found that atypical organ orientation can contribute to diagnostic uncertainty and emphasized the importance of radiologic evaluation when anatomic abnormalities are present [3]. Similarly, systematic reviews of laparoscopic cholecystectomy have demonstrated that surgery in situs inversus is feasible but technically challenging because of altered anatomy and the need for modified operative positioning and port placement [5].

The present case differs from these isolated surgical presentations because the patient underwent a prolonged sequence of abdominal interventions rather than a single procedure. This distinction is particularly relevant in patients requiring repeated imaging and image-guided procedures. The accurate identification of anatomic relationships is important when evaluating fluid collections, fistulous tracts, and drain position. In patients with situs inversus, unfamiliar organ relationships may complicate the interpretation of imaging if the underlying anatomy is not explicitly recognized. Contemporary reviews emphasize the importance of detailed preoperative imaging and anatomic assessment in patients with situs inversus undergoing surgical or interventional procedures [1,2].

The presence of abnormal anatomy is also particularly relevant during revisional bariatric surgery. Bariatric procedures already involve the substantial alteration of gastrointestinal anatomy, and subsequent reoperation may be further complicated by adhesions, altered bowel configuration, fistulas, and previous anastomoses. Biliopancreatic diversion with duodenal switch is a technically complex bariatric procedure involving the substantial rearrangement of the gastrointestinal tract, and careful perioperative management and long-term nutritional follow-up are important components of care [7]. Imaging after duodenal switch also requires familiarity with the expected postoperative gastrointestinal anatomy, as bowel obstruction and anastomotic leaks are among the recognized postoperative complications of the procedure [8]. In addition, the revision of a duodenal switch may be required for postoperative or long-term complications and can involve technically complex operative management [9].

A systematic review of bariatric and metabolic surgery in patients with situs inversus identified 46 reported patients and described the technical considerations associated with altered visceral orientation during bariatric procedures [6]. The literature, however, remains limited, and most reported procedures have involved primary bariatric operations rather than complex revisional surgery with prolonged postoperative complications. The combination of altered intestinal orientation with a previously modified gastrointestinal tract therefore appears to be less extensively described in the literature.

Importantly, the present case does not establish that partial situs inversus caused the patient's postoperative complications. Abscess formation, bowel perforation, enterocutaneous fistula, polymicrobial infection, malnutrition, and the need for repeated surgery are all recognized complications that can occur independently of situs abnormalities, particularly after complex revisional abdominal surgery. Rather, the significance of partial situs inversus in this case lies in its presence as an additional anatomic consideration during an already difficult clinical course.

Another important consideration is the distinction between situs inversus and intestinal malrotation. Although both arise from abnormalities of embryologic left-right development and may coexist, they represent distinct anatomic entities [1,3]. Situs inversus should therefore not automatically be interpreted as evidence of intestinal malrotation. Instead, the patient's actual anatomy should be established through prior imaging and operative findings. In complex surgical patients, discrepancies between expected anatomy and imaging or operative findings should prompt the careful reassessment of the patient's known anatomic configuration.

The case therefore emphasizes that the clinical significance of partial situs inversus may extend beyond the initial recognition of an unusual anatomy. In patients undergoing prolonged and repeated abdominal interventions, the recognition of altered intestinal orientation and careful attention to anatomic relationships may support consistent interpretation and procedural planning. Although partial situs inversus cannot be established as the cause of this patient's postoperative complications, it remained an important anatomic consideration during an already complex revisional bariatric surgical course.

## Conclusions

Partial situs inversus is usually asymptomatic but may require additional consideration in patients undergoing extensive abdominal surgery. In the setting of revisional bariatric surgery, where altered gastrointestinal anatomy, adhesions, postoperative collections, fistulas, and repeated procedures may already complicate care, abnormal visceral orientation represents an additional consideration for imaging interpretation, operative planning, and procedural management.

This case highlights the importance of recognizing altered intestinal anatomy before subsequent interventions and maintaining careful attention to anatomic relationships throughout a prolonged surgical course. Although partial situs inversus cannot be established as the cause of this patient's postoperative complications, it remained an important anatomic consideration during an already complex revisional bariatric surgical course.
